# Stem cells in intervertebral disc regeneration–more talk than action?

**Published:** 2021-12-15

**Authors:** Petra KRAUS, Ankita SAMANTA, Sina LUFKIN, Thomas LUFKIN

**Affiliations:** 1 Department of Biology, Clarkson University, Potsdam, NY 13699, USA; 2 The Clarkson School, Clarkson University, Potsdam, NY 13699, USA

**Keywords:** Stem cells, MSC, Intervertebral disc (IVD), Exosome, Extracellular matrix (ECM)

## Abstract

Pain and lifestyle changes are common consequences of intervertebral disc degeneration (IVDD) and affect a large part of the aging population. The stemness of cells is exploited in the field of regenerative medicine as key to treat degenerative diseases. Transplanted cells however often face delivery and survival challenges, especially in tissues with a naturally harsh microniche environment such as the intervertebral disc. Recent interest in the secretome of stem cells, especially cargo protected from microniche-related decay as frequently present in degenerating tissues, provides new means of rejuvenating ailing cells and tissues. Exosomes, a type of extracellular vesicles with purposeful cargo gained particular interest in conveying stem cell related attributes of rejuvenation, which will be discussed here in the context of IVDD.

## Introduction

Tissues and organs of the aging human body originate from a fertilized oocyte. As this totipotent single cell zygote embarks on its journey of life, most daughter cells will succumb to terminal differentiation eventually followed by death (Kraus and Lufkin, 2017). A few, so called stem cells, retain their potential to divide along with a degree of multipotency (Pittenger *et al*., 1999; Lander, 2009; Sng and Lufkin, 2012). Harnessing or blocking “stemness” is an intriguing approach taken by the fields of regenerative medicine and oncology alike to replenish ailing tissues and organs such as a degenerating intervertebral disc (IVD) or to stop malignant cell growth (Sng and Lufkin, 2012; Sivakamasundari and Lufkin, 2013; Kraus *et al*., 2017; Li *et al*., 2019). Regenerative attempts include transplantation of embryonic stem cells (ESC), induced pluripotent stem cells (iPSC) and transdifferentiated somatic cells (TDSC) (Jopling *et al*., 2011; Xia *et al*., 2019; Wang *et al*., 2021), all however pose risks of tumorigenesis. Furthermore, iPSC and TDSC might be impractical and uneconomical if derived de-novo for each patient (Kamao *et al*., 2014); ESC bear ethical concerns and TDSC are ideally based on detailed knowledge of interacting signaling pathways, which is still lacking for most vertebrate cell types. Autologous or allogeneic mesenchymal stem cells (MSC) however might take in cues from their environment to mold their multipotent potential into the necessary cell type, or, as recently demonstrated, send cues to residual stem cell populations to refurbish ailing tissue (Chen *et al*., 2010; Sun *et al*., 2020; Zhang *et al*., 2020; Luo *et al*., 2021).

### Do MSC live up to expectations?

As of mid-July 2021, a searchable database for worldwide clinical trials lists 1292 hits for “mesenchymal stem cell” including a wide range of treatments such as acute organ failure, stroke, autoimmune disease, diabetes, arthritis, bone fracture, congenital disease, respiratory distress syndrome, cancer and more, with 77 studies currently listed in phase 3 clinical trials (https://clinicaltrials.gov).

As an example, a healthy IVD is crucial for normal spine function where it acts as a semi-moveable joint and provides means of shock absorption to protect adjacent vertebral bodies (Humzah and Soames, 1988; Christ and Wilting, 1992). IVDs are composed of a hydrogel-like, inner nucleus pulposus (NP) rich in extracellular matrix (ECM) that is encapsulated in the annulus fibrosus (AF) and sandwiched by cartilaginous endplates (CEP) (Bibby *et al*., 2001; Sivakamasundari and Lufkin, 2012; Sivakamasundari and Lufkin, 2013). Cells residing in the avascular, non-innervated NP are sparse in a large amount of ECM and depend on diffusion for survival and communication (Urban *et al*., 1977; Urban *et al*., 2004; Binch *et al*., 2015). This creates a niche low in nutrients, oxygen and pH fueled by anaerobic lactic acid fermentation (Urban *et al*., 2004; Wuertz *et al*., 2008; Liang *et al*., 2012). Severe and chronic low back pain (CLBP) caused by age-related IVDD presents a huge socio-economic burden worldwide (DePalma *et al*., 2011; GBD, 2018). Classic treatments of IVDD symptoms are surgical such as discectomy or non-surgical like physiotherapy combined with pain relieving medication (Raj, 2008), the latter contributing to an already overwhelming opiate crisis (Film, 2020). Bioengineering strategies aim for disc repair with injectable hydrogels or replacement with implanted synthetic or natural scaffolds such as polyethylene glycol or alginate, respectively, amongst many others as reviewed in (van Uden *et al*., 2017) sometimes seeded with cells or supplemented with growth factors (Kim *et al*., 2020). The IVD is a welcome target for regenerative approaches as IVDD symptoms typically develop over time without posing an immediate life-threatening situation. This permits the establishment and screening of autologous or allogeneic cell lines for disc refurbishment (Baksh *et al*., 2004; Xu *et al*., 2017). Delivering notochord (NC) cells, healthy NP cells or stem cells to an ailing disc could address IVDD at its roots (McCann *et al*., 2011). In recent years much hope was placed on the injection of MSC with many ongoing clinical trials nearing completion, aiming to increase ECM content of aging discs to restore original disc height. Of 385 ongoing studies listed for IVDD 34 apply stem cells in some form. Earlier clinical studies involving MSC as reviewed in Sakai and Schol (2017) reported pain relief and increased disc hydration but no improved disc height. A study involving reactivated NP cells after MSC coculture achieved pain relief without deteriorating disc height (Blanco *et al*., 2010). More recently, a study sponsored by Bioheart, Inc. using adipose stem cells to assess safety and efficacy has pending results despite completion in 2017 (NCT02097862). Recent phase 2 data for the Mesoblast sponsored CASCADE phase 3 clinical trial using the mesenchymal precursor cell-based product Rexlemestrocel-l for CLBP suggests a potentially safe and effective treatment (Kraus and Lufkin, 2017; Amirdelfan *et al*., 2021). NOVOCART^®^ disc, an active phase 1/2 study investigating an autologous disc chondrocyte transplantation system sponsored by Tetec AG is expected to post results soon (NCT01640457) (Tschugg *et al*., 2017; Li *et al*., 2019). However, as straight forward as stem cell based approaches might seem, the microenvironment in the aging disc is harsh and presents an obstacle for cell survival, either native or transplanted (Sivakamasundari and Lufkin, 2013). While data from preclinical animal models showed promising results in restoring a disc phenotype (Sakai *et al*., 2003; Crevensten *et al*., 2004; Sakai *et al*., 2005; Henriksson *et al*., 2009), whether MSC live up to their expectations in IVDD therapy will depend on the outcome of further clinical trials and their long term follow up.

### Exosomes–a way of “talking” long distance?

**FIGURE 1.**
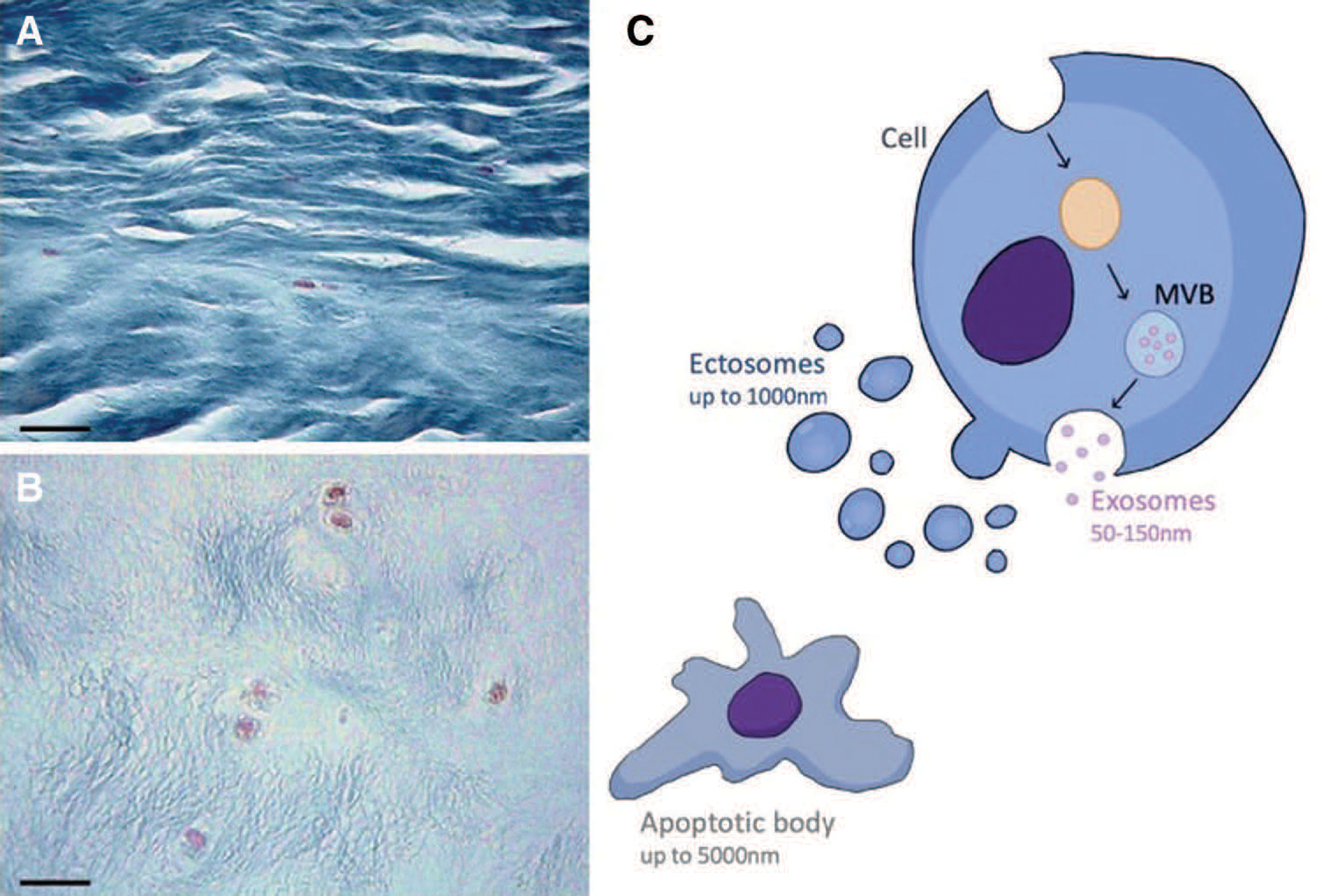
Mallory’s tetrachrome stain visualizes that cell nuclei (magenta) of the annulus fibrosus (A) and nucleus pulposus (B) of a mature bovine IVD are sparse in a vast amount of extracellular matrix (blue) requiring long-distance communication of some kind. Bar = 50 μM. (C) Extracellular vesicles (EV) are generated by apoptosis (apoptotic bodies) through membrane budding (ectosomes) or the endosome pathway (exosomes). Exosomes are released into the extracellular environment when multivesicular bodies (MVB) fuse with the plasma membrane.

Homeostasis of a microenvironment is naturally maintained through effective cell-cell communication. IVD cells are sparsely embedded in a large amount of ECM as presented in Figs. 1A and 1B. NP cells reside in an avascular, non-innervated environment as presented in Fig. 1B requiring other communication skills (Liebscher *et al*., 2011; Li *et al*., 2019; Li *et al*., 2019). Development of large scale “-omics” technologies studying proteins as cells release them into their environment (the secretome) increased our understanding of cell-cell communication, enabling the study of extracellular vesicles (EV) with purposeful cargos of proteins and nucleic acids (Jeppesen *et al*., 2019). EVs differ in size and origin: Apoptotic bodies (50 nm–5000 nm) and ectosomes (50 nm–1000 nm) are generated through outward budding of the plasma membrane, while exosomes (50 nm–150 nm) are generated through the endosomal generation of multivesicular bodies (MVB) (Kowal *et al*., 2016) as presented in Fig. 1C. Exosomes are generated by most cell types (Edgar, 2016; Kalluri and LeBleu, 2020) and their release into body fluids as well as culture media, generates interest for cancer biomarker identification (Kalluri, 2016; Couto *et al*., 2018).

The NP is of NC origin (Christ and Wilting, 1992; Choi *et al*., 2008; Choi and Harfe, 2011; Choi *et al*., 2012; McCann *et al*., 2012; Lawson and Harfe, 2015; McCann and Seguin, 2016). Progressive loss or trans-differentiation of NC cells in humans and other species like *Bos taurus* coincides with the onset of IVDD, while the adult murine NP remains composed of NC cells (Trout *et al*., 1982; Urban and Roberts, 2003; Vujovic *et al*., 2006; Gilson *et al*., 2010; Kraus *et al*., 2017). Coculture of NC cells with MSC or the use of conditioned NC medium could transform MSC towards a NP-like phenotype (McCann *et al*., 2011; Purmessur *et al*., 2011 and recently, bone marrow derived MSC could be differentiated towards a NC phenotype through culture with pulverized porcine NP matrix (Li *et al*., 2021). Secreted signaling factors likely mediate these effects *in vitro* (Yang *et al*., 2008; Strassburg *et al*., 2010; Ferreira *et al*., 2021). However, the large distance these factors must travel in the NP-ECM puts them at risk for degradation prior to reaching a target cell. The exosome phospholipid-bilayer would provide necessary protection for signaling molecules. Exosomes isolated from NC conditioned medium showed similar transforming properties (Sun *et al*., 2020) and the described bidirectional exchange of membrane components *via* multisize vesicles during NP cell and MSC coculture (Strassburg *et al*., 2012) supports such mechanisms. While the key-proteome of exosomes was recently identified (Kugeratski *et al*., 2021), exosome cargo can be specific to the cell line of origin and trigger diverse outcomes in target cells (Edgar, 2016; Kalluri and LeBleu, 2020). But can exosomes travel through the dense ECM meshwork *in vivo*? It seems theoretically possible (Lenzini *et al*., 2020). An ongoing clinical trial sponsored by the Dr Himanshu Bansal Foundation using platelet derived exosomes to treat IVDD might provide *in vivo* practical evidence by Spring 2022 (NCT04849429).

### Future outlook

Many cells receive critical cues from the ECM. Mimicking these conditions *in vitro* requires elaborate hydrogels or scaffolds and even mechanical cues on top of media supplements. Hence maintaining a cell’s phenotype in culture can be more challenging than isolating it. Achieving critical cell numbers for therapy through expansion creates a dilemma between practical 2D and 3D culture, the latter being required for phenotypical identity. Recently, many of the attributes of MSC in tissue regeneration are projected on cell-cell communication or “talking” of MSC to endogenous stem cells through means such as exosome cargo instead of “action” in the form of homing and replication of MSC in the target tissue (Richardson *et al*., 2016; Croft *et al*., 2021). If exosome application can replace cell-transplantation, conditioned medium could be harvested from cells maintained in 3D culture, minimizing therapeutic cell loss in non-permissive endogenous environments or through immunogenic rejection.

Cells in the IVD find themselves in the unique situation of being very distant from their neighbors without the usual lifelines of communication (Vadala *et al*., 2019). In that context it seems plausible that peptides, proteins, and nucleic acids get deposited into vesicles like exosomes for a protected journey. The exosome concept might be further exploited in IVD therapies (Piazza *et al*., 2020) with natural or synthetic exosomes loaded with therapeutic cargo for safely delivering anti-inflammatory cytokines, transcription factors, growth factors and means to regulate metalloproteases. Such exosome focused therapies could circumvent some of the problems associated with stem cell transplantation such as rejection or tumor formation and reduce stem cell tourism due to national laws currently restricting many stem cell therapies in the US, Canada and Europe for ethical or safety concerns (Master and Resnik, 2011; Brown, 2012). As an example, the use of embryonic stem cells is seen as unethical by many, while harvesting exosomes from conditioned medium might receive less criticism. Lastly, a long ongoing quest for biomarkers defining IVD cell populations (Gilson *et al*., 2010; Minogue *et al*., 2010; Minogue *et al*., 2010; Risbud *et al*., 2015; Thorpe *et al*., 2016; Richardson *et al*., 2017; van den Akker *et al*., 2017; Kraus *et al*., 2019; Li *et al*., 2019; Li *et al*., 2019; van den Akker *et al*., 2020) might be addressed through the analysis of exosome cargo, just as it has been done in the field of cancer diagnostics (Makler and Asghar, 2020).

## Abbreviations

AF: Annulus fibrosus
CEP: Cartilaginous endplates
CLBP: Chronic low back pain
ECM: Extracellular matrix
ESC: Embryonic stem cells
EV: Extracellular vesicles
iPSC: Induced pluripotent stem cells
IVD: Intervertebral disc
IVDD: IVD degeneration
MSC: Mesenchymal stem cells
MVB: Multivesicular bodies
NC: Notochord
NP: Nucleus pulposus
TDSC: Transdifferentiated somatic cells
